# Establishing Diagnostic Reference Levels in Digital Mammography from Eight Mammography Units Using over 30,000 Images

**DOI:** 10.3390/diagnostics15060682

**Published:** 2025-03-10

**Authors:** Mirjeta Mediji-Arifi, Mimoza Ristova

**Affiliations:** 1 Physics Department, Faculty of Natural Sciences and Mathematics, State University of Tetovo, 1200 Tetovo, North Macedonia; mirjeta.ma@gmail.com; 2 Physics Department, Faculty of Natural Sciences and Mathematics, University Ss Cyril and Methodius, 1000 Skopje, North Macedonia

**Keywords:** digital mammography, mean glandular dose (MGD), compressed breast thickness, diagnostic reference level (DRL), radiation exposure

## Abstract

**Introduction:** Diagnostic reference levels (DRLs) in digital mammography were determined from 31,040 digital mammography images acquired from diagnostic and screening examination data from eight state-managed mammography centers/units in the Republic of North Macedonia (RM). The main objective is to establish a diagnostic reference level for mammography examinations at different ranges of breast thickness. **Materials and methods**: Approximately 30,000 mammography images were used to evaluate mean glandular dose (MGD) and compressed breast thickness (CBT) for each projection, craniocaudal (CC) and mediolateral oblique (MLO). The stratified DRL was derived by calculating the 75th percentile of the MGD across all the samples at various CBT ranges for both projections. **Results and Discussion**: The overall median MGDs, minimum, and maximum were calculated to be 1.15 mGy, 0.1 mGy, and 9.93 mGy, respectively. As the CBT increased from 7 to 120 mm, the 75th percentile of the MGD increased from 0.94 mGy to 3.67 mGy for CC, and from 0.44 mGy to 4.91 mGy for MLO projections. **Conclusions**: The study established local DRLs for the digital mammography systems at the 75th percentile, which compared well with the values reported for other countries/regions. The DRL defined per CC and MLO image view for a specific CBT indicated that at least one mammography facility needs optimization.

## 1. Introduction

Mammography diagnostics and screening examinations are very important procedures for early breast cancer detection, which was proven to minimize mortality rate by 25–40% [1]. Estimating the absorbed dose delivered to the breast is a crucial aspect of quality control in mammography examinations, as there exists a small but significant stochastic risk of radiation-induced breast cancer associated with the X-ray exposure from this procedure [2,3,4]. One of the crucial parameters for measuring the X-ray exposure is the mean glandular dose (MGD), defined as the average energy deposited in all breast glandular tissues per unit mass of the glandular tissue. Furthermore, the International Commission on Radiological Protection (ICRP) suggested that MGD within the breast is the most convenient quantity [5]. It is important to emphasize that the MGD calculations are made under the assumption that the breast tissue is homogeneous [6]. The radiation dose delivered to the breast can be calculated in different ways through the screening, and it depends on the breast granularity, breast thickness, tube voltage (kVp), energy spectrum depending on the target–filter combination, and the compression force (CF). The estimation of MGD can be achieved by utilizing either a standard patient or a standard phantom [7].

The increase in the compression force (CF) is aimed at decreasing the breast thickness and reducing radiation dose [8], thereby allowing a mammography examination to give accurate diagnostic information at the same time as delivering an acceptable dose to the breast, without increasing the potential risk of causing radiation-induced cancer [9].

To improve dose optimization in radiological examinations, the International Commission on Radiological Protection (ICRP) set recommendations on the use of diagnostic reference levels (DRLs) [10], defined as a dose level for a typical X-ray examination of a group of patients of standard body size, using broadly defined types of digital mammography equipment. These levels are expected not to be higher for standard procedures when good and normal practice is applied, depending on the diagnostic and technical performance [11]. The introduction of the DRL parameter provides an indicator of quality monitoring to limit the variation in the dose among different imaging centers/units [10]. Again, the DRLs are the most common parameter used for quality control, comparison of dose levels, optimization, and limitation of differentials in dose among mammography imaging centers (units in the preset work).

So far, North Macedonia does not have a national/regional registry of DRLs in mammography. The typical values in mammography units were established with the present study, as a first step towards raising the need for optimization. Diagnostic reference levels (DRLs) were introduced for quality control, optimization, and protection [12] to help limit variations in dose delivered among and within imaging centers and these levels are expected not to be exceeded for a standard diagnostic procedure when good and normal practice is applied [13].

## 2. Materials and Methods

The data for the present study were taken from eight mammography units in North Macedonia (NM). Having in mind that different technologies were used in this study (as shown in Table 1), i.e., Fuji in seven mammography units and Hologic Selenia in one unit, the data acquisition process was heterogeneous. Herein, the organ dose data from Fuji mammographs in Units A, B, C, D, E, F, and G were obtained from DICOM headers using dose monitoring software (DOSE, v.17.11, Qaelum NV, Leuven, Belgium). However, in the last unit, Unit H, dose values were extracted from DICOM and exported as *.csv format files using the open-source application ‘Micro DICOM Viewer’. All the mammography unit management teams confirmed that the data had been verified through internal weekly image quality assurance procedures conducted by technical personnel using mammography phantoms, as well as through an annual external quality control audit, performed by the Directorate of Radiation Safety of the Republic of North Macedonia. Herein, the variation in data acquisition methods is expected to represent only a minor limitation since previous research has reported an estimated variation within 10% [14] among MGDs calculated using three specific dose models (Boone, Dance, and Wu) adopted by different vendors.

The eight mammographical units for this study were selected from the total of twenty-five, representing 32% of all the digital mammography units in in North Macedonia [15] with even geographical representation. All the full digital mammography images were taken of cases (only women) aged between 10 and 89 years in both the breast screening and diagnostic procedures. Each examination consisted of taking a total of 4 projections: right and left breast craniocaudal (CC) view and right and left breast mediolateral oblique (MLO) view. There were a total of 31,040 mammographic projections (7760 × 4 positions = 31,040). The manufacturer and relevant technology details of the digital mammography devices in each unit are listed in Table 1.

The data acquired from the DOSE and DICOM were then extracted and transferred to Microsoft Office Excel for further numerical analysis. The data for each mammography unit were categorized according to their compressed breast thickness in intervals of 10 mm. The mean glandular dose (MGD) values per mammography unit were calculated for each thickness interval. The following main statistical data were calculated: minimum dose (mGy), maximum dose (mGy), median dose (mGy), standard deviation, mean thickness (mm).

The compression force (CF) applied to the breast during mammography was nominally sufficiently high to ensure the thickness is no longer a limiting factor for visualizing details, such as microcalcifications in the breast, but sufficiently low to only induce tolerable pain for most women. Also, the optimal force should adequately thin the breast, while not causing excessive distortion of the anatomic features.

From all the MGDs calculated for all projections, we have: (a) evaluated the 75th percentile from all the mammography units in RM and compared them to the results reported for other countries and applied the same methodology (i.e., patient survey); (b) compared the mean MGD value to the European dose levels for screening and diagnostic mammography procedures for compressed thicknesses (CBTs) ranging from 7 to 119 mm; (c) compared the mean MGD for each of the eight mammography units for each CBT (range between 30 mm and 75 mm) with European achievable dose levels, European acceptable dose levels, and Belgian diagnostic reference levels. Herein, the acceptable dose level (ACC) is the maximum mean value of MGD, while the achievable dose level (ACH) is the recommended level [16,17].

According to the European Guidelines [18], the acceptable dose level (ACC) represents the highest mean glandular dose (MGD) for the equivalent breast, while the achievable dose level (ACH) indicates the recommended operating standard for the mammography systems. According to European guidelines, both the acceptable and achievable levels are established through calculations based on European formalism, utilizing conversion coefficients developed by Dance et al. [19,20].

All the transferred data were then analyzed using SPSS version 25. Correlations between CBT, patient age, compression force, tube voltage, tube current, and entrance dose for both MLO and CC were analyzed using the Pearson correlation coefficient test. For a non-normal distribution dataset, the parametric one-way ANOVA test was used. Descriptive statistics were used to acquire MGD mean, median, standard deviation, percentiles, and range. The differences among MGDs across different CBT groups were tested using the Kruskal–Wallis test.

Finally, the research was conducted under strict ethical considerations and the information and identity of the contributors with their mammograms were confidential and used only for the present study by restricting access to the study information which was in hard copy as well as soft copy documents with password protection. Anonymity was maintained for all eight participating state hospitals/units and study participants by using alphabetical letters (A, B, C, D, E, F, G, and H) and serial numbers for hospitals and patients rather than their names, respectively.

## 3. Results and Discussion

**Figure 1:**
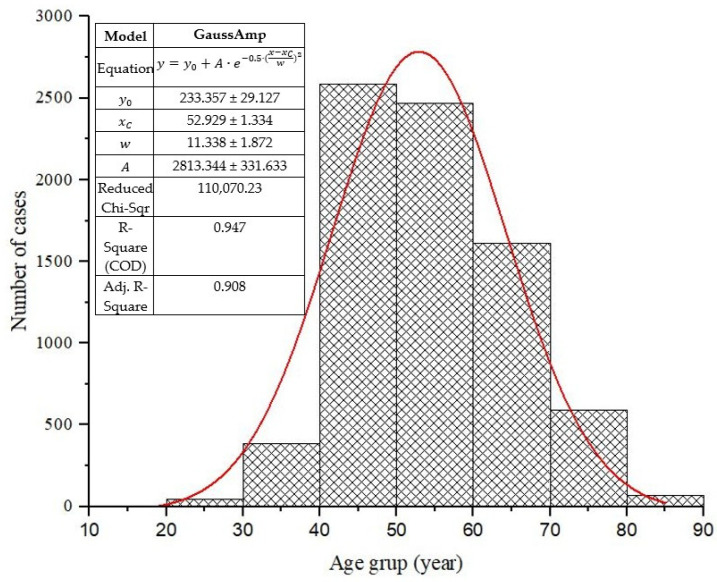
Normal age distribution of the mammography cases (total number 7760) subject to this study.

The study included 7760 women who underwent mammography examination, ranging between 10 and 89 years with a mean age of 54.12 ± 11.5 years, as presented in Figure 1. The cases were organized into age groups (step of 10 years) from 10–19, 20–29, 30–39, …, and 80–89 years.

The normal age distribution of the cases for diagnostic and screening examinations is presented in Figure 1. Herein, according to the Gaussian fit (red line), the age group 50–59 years occurs with the highest frequency, representing about 33% of cases, while the age group of 10–19 years recorded the lowest number of patients, representing 0.04% of reported cases.

In Table 2 we present the overall summary of the background data taken from each unit: number of images taken from each mammography unit, number of cases examined, mean age per unit, median MGD per image, view (MLO, CC), compressed breast thickness, kVp, and mAs. The table also includes the mean patient age and exposure time. The evaluated mean MGD/view for all patients for each MLO and CC image were calculated to be 1.34 mGy and 1.61 mGy, respectively. Examination of the data in Table 2 demonstrated minimal differences in the calculated mean doses between the CC and MLO projections.

In Table 3 we present the relevant patient characteristics and scanning parameters across ranges of breast thicknesses, for both MLO and CC projections. From the data in Table 3, notably, with the increase in the compression force (CF), X-ray tube voltage, and tube current, the entrance dose also increased for both MLO and CC projections.

The dependence of the mean MGD on CBT is represented by curves fitted using an exponential function based on the least squares method applied to the empirical data points. For comparison purposes, we also present the mean MGD versus CBT curves alongside the acceptable European, achievable European, and Belgian diagnostic reference levels, as described in the European guidelines for screening and diagnostic mammograms [16,17,18,19].

The results of the MLO projections reveal the following Pearson correlation findings: there is an insignificant negative relationship between thickness (mm) and patient age (r = −0.0789), as well as an expected insignificant negative relationship between thickness (in mm) and compression force (in N) (r = −0.068). Additionally, a significant large positive relationship is observed between thickness (mm) and tube voltage (kV) (r = 0.857), a significant medium positive relationship between thickness (mm) and tube current (mAs) (r = 0.331), and a significant large positive relationship between thickness (mm) and entrance dose (mGy) (r = 0.535). Similarly, for the CC projections, comparable trends are observed. The Pearson correlation indicates an insignificant negative relationship between thickness (mm) and patient age (r = −0.116), as well as an insignificant negative relationship between thickness (mm) and compression force (N) (r = −0.0801). However, there is a significant large positive relationship between thickness (mm) and tube voltage (kV) (r = 0.847), a significant medium positive relationship between thickness (mm) and tube current (mAs) (r = 0.329), and a significant large positive relationship between thickness (mm) and entrance dose (mGy) (r = 0.515) (Table 3).

The results of the one-way ANOVA revealed significant differences among the imaging parameters across all CBTs. As the CBT increased, the compression force applied showed a significant increment (*p* < 0.001), suggesting that greater force is required to achieve optimal image quality in thicker breasts. Similarly, we observed statistically significant increments in the tube voltage (*p* < 0.001) and tube current (*p* < 0.001) with the increase in the CBT. Additionally, the entrance dose demonstrated a significant rise (*p* < 0.001) with the increase in breast thickness (Table 3).

**Figure 2:**
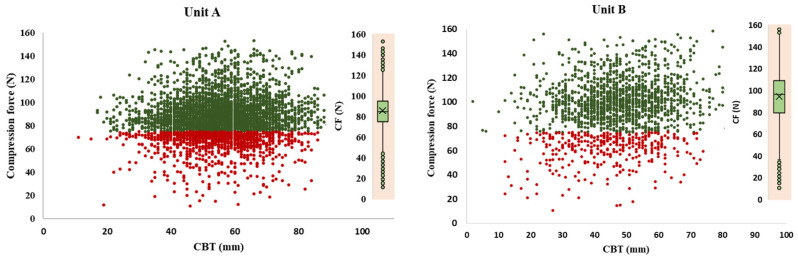

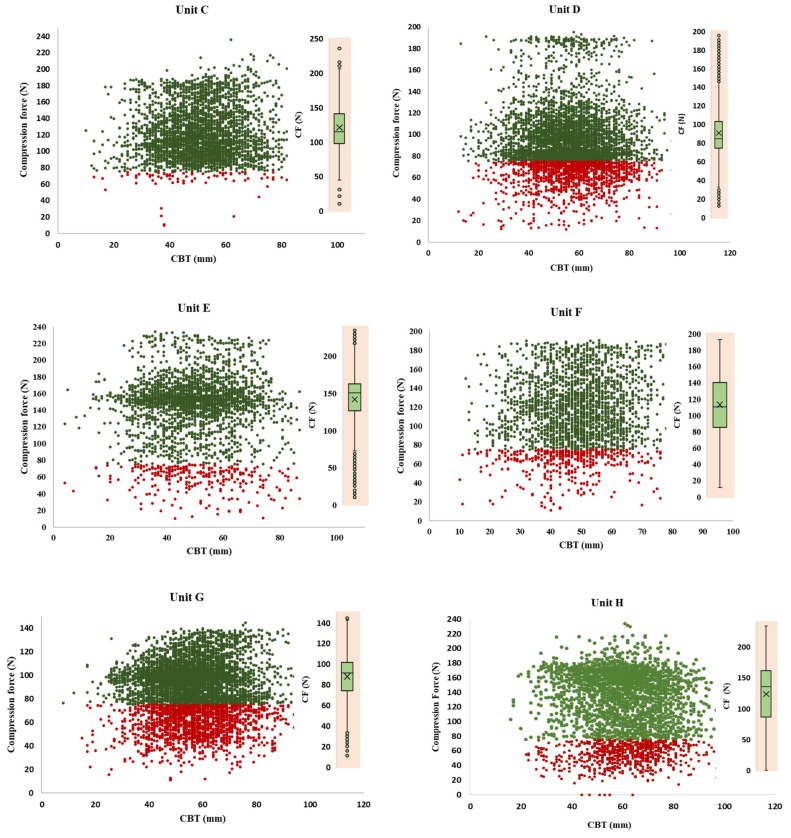
Compression Breast Thickness (CBT in mm) as a function of the compression force (CF in N) for all projections across the eight examined Mammography Units A, B, C, D, E, F, G, and H in North Macedonia. Red labels represent lower compression forces than the recommended and green ones, higher than the recommended.

The distributions of the compression breast thickness (CBT), with a mean compression force (CF) for the eight mammography units, are depicted in Figure 2. Herein, red data labels represent cases of lower compression force than the recommended one (<75 N), while the green ones present those above the recommended values. All eight charts in Figure 2 clearly show that a significant number of patients (red dots) experienced compression forces much lower than the recommended value. The cases represented by the green dots experienced compression that exceeded the recommended value. It is evident that the radiographers in Unit C with a mean CF of 119.13 ± 34.35 N, Unit E with 140.90 ± 41.02 N, and Unit F with 112.45 ± 37.90 N applied significantly higher compression forces in comparison with the remaining units, Unit A with mean CF 84.40 ± 20.19 N, Unit B with 94.17 ± 23.88 N, Unit D with 90.22 ± 29.31 N, Unit G with 86.53 ± 24.416 N, and Unit H with with 123.54 ± 44.58 N.

**Figure 3:**
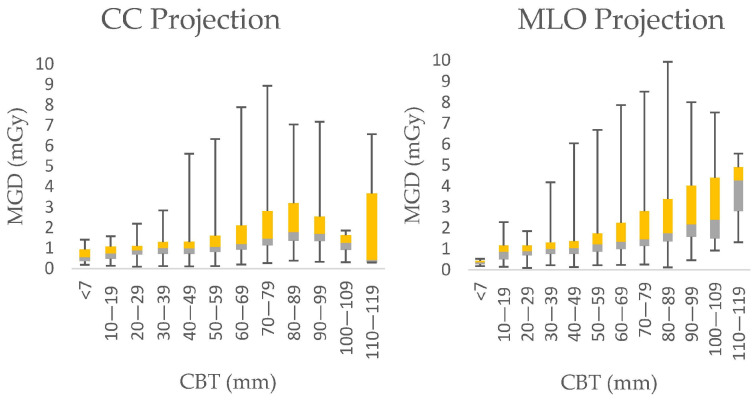
Boxplots presenting the minimum dose (mGy), maximum dose (mGy), median dose (mGy), 25th percentile and 75th percentile of MGD for: (**left**) MLO and (**right**) CC projections in different CBT groups, evaluated from all the data of the eight mammography units in North Macedonia. The bordelines between the yeallow and the grey boxes represent the median of MGD, while the yellow box demonstrates the interquartile range from median to 75th percentile, and the grey one, from median to the 25th percentile.

The error bars from Figure 3 stretch between the minimum and maximum MGD range, the line within each rectangular box represents the median MGD value (i.e., DRL value), while the rectangular box’s upper and lower borders demonstrate the interquartile range (IQR: 25th to 75th percentile). The median MGD ranges between 0.56 mGy and 1.78 mGy for CC and between 0.36 mGy and 4.28 mGy for MLO. Also, it appeared that the maximum MGD values for each 10 mm CBT interval range between 1.41 mGy and 8.94 mGy for CC and between 0.53 mGy and 9.93 mGy for MLO. 

Furthermore, the Kruskal–Wallis test showed a statistically significant difference between the groups (*p* < 0.001). At the 75th percentile, the measured parameter values are 0.94, 1.08, 1.11, 1.3, 1.61, 2.11, 2.81, 3.2, 2.54, 1.63, and 3.67 mGy for CC and 0.44, 1.17, 1.32, 1.38, 1.75, 2.25, 2.80, 3.38, 4.02, 4.40, and 4.91 mGy for MLO projections in the respective CBT ranges of <9 mm, 10–19 mm, 20–29 mm, 30–39 mm, 40–49 mm, 50–59 mm, 60–69 mm, 70–79 mm, 80–89 mm, 90–99 mm, 100–109 mm, and 110–119 mm. The 75th percentile values, which are considered to be local diagnostic reference levels, exhibit an increasing trend for the higher CBT ranges, indicating a potential correlation between the measured parameter and breast thickness (Figure 3). 

The extracted MGDs for the reported national levels are listed in Table 4, showing variations across the nation’s DRLs. In our study, the 75th percentile of the MGD increases from 0.94 mGy and 3.67 mGy for CC and from 0.44 mGy to 4.91 mGy for MLO, depending on the increase in the breast thickness. A New South Wales study also presents results for digital radiography (DR) and computed radiography (CR), for CBT intervals of 20 mm to 110 mm. The latter study shows similar results for CBT to those of our present study, although they have considered different detector technologies. However, in the present study, for comparison we consider only results from DR systems (9). The comparison showed that the respective MGD (9) spreads within the range from 0.97 to 3.31 mGy, which is generally higher than ours, except for those of CBT lower than 30 mm. Furthermore, the results from Ghana by Dzidzornu et al. [21] present the 75th percentile of MGD for CC projections to be 1.6 mGy (CBT: 36 mm) and 2.4 (CBT: 45 mm) for MLO projections, which appear to be higher than those of our present study. Also, as evident from a local report for Malaysia, the DRLs for CC and MLO are 1.68 mGy (CBT: 50.9 mm) and 2.25 mGy 2.25 (CBT: 58.9 mm), respectively, showing values generally higher than ours [22]. In Sudan, the local DRLs for DR were 3.48 mGy (CBT: 36 mm) for CC and 2.03 mGy (CBT: 44 mm) for MLO [23], and the figures show that the calculated DRLs are lower than those registered in Gaza (2.5 mGy, 50–60 mm for CC projections) [24]. Furthermore, the mean CBT value recorded in Morocco is 55 mm. The DRLs reported for all three diagnostic centers are 1.6 mGy, 1.7 mGy, and 1.8 mGy [25]. Notably, the values are close to those evaluated in our study. Finally, in our study, we recorded higher DRL values compared to the two studies performed in Dubai [26,27]. The higher DRLs could be due to the different procedures and techniques employed in their studies.

Table 5 presents typical MGD (mGy) values for diagnostic and screening mammography for CC and MLO projections along with European DRLs [18]. Our typical values were found to be lower than the acceptable European values and also lower than the achievable European values.

**Figure 4:**
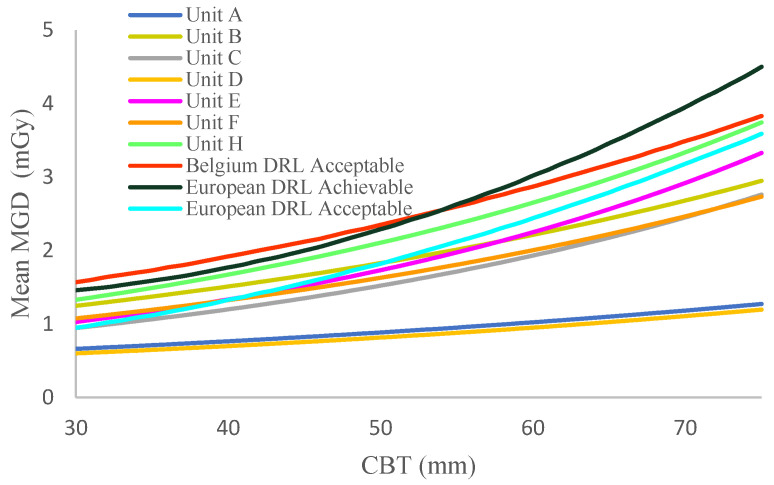
Mean MGD (mGy) against the CBT (mm). The curves of Units A, B, C, D, E, F, G, and H are flattened by fitting with exponential functions with least square roots. European reference curves (Belgian and EU) are added for comparison.

In Figure 4 we present the dependence of the average mean glandular dose (MGD) values obtained from craniocaudal (CC) and mediolateral oblique (MLO) values of the compression breast thickness (CBT) within the range between 30 mm and 75 mm for eight units (A, B, C, D, E, F, G, H) in North Macedonia. As evident from Figure 4, the mean glandular dose (MGD) values for North Macedonia for seven of the eight examined (out of a total of twenty-five) mammography units were lower than the European achievable dose level (ACH), the European acceptable dose level (ACC), and Belgian diagnostic reference levels. However, the results from Unit H appeared to be slightly higher than the European ACC. These differences may be attributed to variations in technology. Additionally, slight differences in compressed breast thickness may account for the higher doses observed. These findings highlight opportunities for optimizing practices in each unit.

## 4. Conclusions

Dose reference values were evaluated from approximately 30,000 digital mammography images from over 8000 diagnostic and screening cases. The data were collected between 2020 and 2024 from eight mammography units in North Macedonia.

Our analysis showed that the mean glandular dose (MGD) from all examinations included in this study, obtained from both the CC and MLO projections of both breasts, were evaluated to be 1.34 mGy and 1.61 mGy, respectively. The median MGD was calculated to be 1.15 mGy, with minimum and maximum values of 0.1 mGy and 9.93 mGy, respectively. The 75th percentile of the MGD for CC acquisitions increased from 0.94 mGy to 3.67 mGy with the rise in compressed breast thickness (CBT) within the range of 0–120 mm. Similarly, the 75th percentile of MGD for MLO acquisitions increased from 0.44 mGy to 4.91 mGy with the increase in CBT.

The MGD values for North Macedonia for seven of the eight examined (out of a total of twenty-five) mammography units were found to be lower than the European achievable dose level (ACH), the European acceptable dose level (ACC), and Belgian diagnostic reference levels. However, the results from Unit H appeared to be slightly higher than the European ACC. Variations in technology and slight differences in compressed breast thickness may account for the higher doses observed in this unit. These findings highlight opportunities for optimizing practices across all units.

A comparative analysis of diagnostic reference levels (DRLs) in mammography of North Macedonia and other countries/regions reveals distinct variations in radiation dose practices and protocols. Our study demonstrates that DRLs in North Macedonia may differ from international benchmarks due to factors such as patient demographics and screening and diagnostic practices but also the variations in technology and equipment. We have also shown that differences in mammography imaging parameters, including compression force and exposure settings, contribute to the observed dose variations in DRLs.

In summary, this study introduces patient-specific diagnostic reference levels (DRLs) for different ranges of compressed breast thickness (CBT) in North Macedonia. The establishment of these values provides valuable insights into the radiation dose associated with screening and diagnostic mammography, supporting the initial efforts for optimization and standardization of mammography procedures for patient safety and imaging quality in the country.

In our future research, we will explore the extracted glandular density data from each image using Laboratory for Individualized Breast Radiodensity Assessment (LIBRA Version 1.0.4), a software tool that quantifies glandular density as a percentage. We will analyze the glandular density distribution across different age groups using statistical methods. Furthermore, we will investigate the influence of the operator/technician in selecting setup parameters, such as compression force (CF), tube voltage, and tube current on the mean glandular dose (MGD), utilizing the available coded technician identity for each case.

## Figures and Tables

**Table 1:** Mammograph manufacturer, technology, anode/filter combinations, number of projections, and patients included in the dose audit from mammography units in NRM.

Unit	Manufacturer	Technology	Anode/Filter	Projections	Cases
A	Fuji Innovality	DR	W/Rh	4520	1130
B	Fuji Amulet s	DR	W/Rh	1772	443
C	Fuji Amulet s	DR	W/Rh	3436	859
D	Fuji Amulet s	DR	W/Rh	5672	1418
E	Fuji Amulet s	DR	W/Rh	2832	708
F	Fuji Amulet s	DR	W/Rh	2608	652
G	Fuji Innovality	DR	W/Rh	6380	1595
H	Hologic Selenia	DR	W/Ag, W/Rh	3820	955

DR: Digital radiography, W: Tungsten, Rh: Rhodium, Ag: Silver.

**Table 2:** Number of images/examinations per mammography unit (A, B, C, D, E, F, G, H), exposure parameter mean, median entrance surface air kerma, and median mean glandular dose per view, image, and case, for 31,040 mammograms across 8 units.

Mammography Unit			A	B	C	D	E	F	G	H	All
No. of cases			1130	443	859	1418	708	652	1595	955	7760
No. of images			4520	1772	3436	5672	2832	2608	6380	3820	31040
Mean Age (years) (StDev)			51.30 (11.50)	55.24 (10.51)	54.80 (10.29)	54.85 (13.24)	55.00 (12.75)	54.89 (12.60)	55.32 (12.67)	51.63 (8.56)	54.12 (11.51)
Mean Thickness (mm) (StDev)			56 (13)	47 (13)	51 (12)	56 (13)	50 (12)	48 (13)	57 (13)	60 (13)	54 (13)
Mean kVp (StDev)			29.34 (1.67)	28.45 (1.35)	28.82 (1.25)	29.41 (1.48)	28.69 (1.37)	28.55 (1.43)	29.53 (1.42)	30.15 (1.84)	29.11 (1.47)
Mean mAs (StDev)			67.74 (30.56)	108.89 (49.33)	95.81 (40.97)	54.24 (17.10)	129.70 (68.66)	102.92 (38.64)	70.60 (22.41)	250.41 (93.11)	110.03 (45.09)
Mean ESAK (mGy) (IQR)			3.54 (1.79)	5.73 (2.78)	5.44 (3.26)	3.28 (1.71)	6.21 (4.2)	5.23 (2.85)	3.56 (1.82)	11.42 (7.98)	3.9 (3.14)
Mean MGD/View (mGy)	CC	R	0.92	1.58	1.47	0.86	1.59	0.93	0.93	2.61	1.36
		L	0.92	1.57	1.40	0.85	1.59	0.92	0.92	2.48	1.33
		R + L	0.92	1.57	1.43	0.85	1.59	0.92	0.92	2.54	1.34
	MLO	R	1.04	1.98	1.79	0.99	2.07	1.06	1.06	2.95	1.61
		L	1.05	1.96	1.80	0.96	2.09	1.06	1.06	2.88	1.60
		R + L	1.04	1.97	1.80	0.97	2.08	1.06	1.06	2.92	1.61
Median MGD (mGy) (IQR)			0.91 (0.39)	1.63 (0.76)	1.47 (0.79)	0.86 (0.36)	1.63 (1.02)	1.50 (0.69)	0.94 (0.38)	2.61 (1.46)	1.15 (0.84)

StDev: Standard deviation; Thickness: compressed breast thickness; IQR: Interquartile range; MGD: Mean glandular dose; ESAK: Entrance surface air kerma; CC: Craniocaudal view; MLO: Mediolateral Oblique view; R: Right breast; L: Left breast; kVp: X-ray tube potential; mAs: X-ray tube current-time product.

**Table 3:** Patient characteristics and scanning parameters across breast thicknesses for MLO and CC projections (n = 31,040).

View	CBT (mm)	N	Thickness (mm)	Age (years)	Compression Force (N)	Voltage (kV)	Tube Current (mAs)	Entrance Dose (mGy)
					Mean ± Std (Range)			
MLO	<7	2	4.5 ± 0.7	88.0 ± 0.0 (88–88)	143.9 ± 28.6 (123.6 –164.1)	23.0 ± 0.0 (23–23)	29.5 ± 19.0 (16–43)	0.5 ± 0.4 (0.3–0.8)
	10–19	59	16.5 ± 2.3	57.3 ± 15.3 (31–86)	96.3 ± 39.5 (17.4–178.4)	25.6 ± 0.9 (22–28)	49.2 ± 24.9 (10–126)	1.6 ± 0.8 (0.3–3.7)
	20–29	318	26.0 ± 2.6	54.2 ± 12.7 (25–91)	104.9 ± 37.2 (0.0–193.1)	26.2 ± 0.6 (24–28)	63.4 ± 29.1 (10–164)	2.1 ± 0.8 (0.3–4.3)
	30–39	1156	35.4 ± 2.8	54.7 ± 12.1 (28–88)	111.2 ± 37.6 (0.0–229.0)	27.6 ± 0.8 (26–30)	75.2 ± 44.4 (13–368)	2.9 ± 1.3 (0.6–11.4)
	40–49	2661	45.0 ± 2.9	54.7 ± 11.4 (25–87)	109.2 ± 36.5 (15.9–233.2)	28.1 ± 0.4 (26–33)	83.6 ± 52.4 (10–454)	3.6 ± 1.8 (0.4–18.8)
	50–59	4226	54.7 ± 2.8	55.1 ± 10.5 (25–90)	108.1 ± 36.0 (0.0–227.8)	29.5 ± 0.9 (26–34)	98.1 ± 63.1 (0–507)	4.8 ± 2.5 (0–24.5)
	60–69	4063	64.2 ± 2.9	53.9 ± 9.6 (27–83)	107.3 ± 36.6 (0.0–235.9)	30.3 ± 0.7 (28–38)	127.1 ± 93.0 (17–574)	6.7 ± 4.2 (0.9–30.4)
	70–79	2093	73.8 ± 2.8	52.8 ± 8.9 (27–86)	104.3 ± 37.2 (10.9–224.1)	31.0 ± 1.0 (28–34)	139.3 ± 89.4 (2–574)	8.7 ± 5.2 (0–35.1)
	80–89	708	83.6 ± 2.9	51.9 ± 8.8 (32–82)	96.4 ± 37.6 (14.0–200.8)	31.9 ± 1.6 (29–34)	160.7 ± 98.3 (0–555)	11.2 ± 7.0 (0–51.6)
	90–99	138	93.3 ± 2.9	51.6 ± 8.0 (29–76)	93.5 ± 34.7 (23.4–192.9)	33.2 ± 1.2 (28–35)	181.1 ± 103.2 (45–509)	14.4 ± 8.1 (2.4–41.2)
	100–109	25	102.6 ± 2.1	52.4 ± 8.7 (43–79)	81.7 ± 36.0 (27.6–156.8)	33.9 ± 2.3 (30–37)	174.5 ± 111.4 (53–428)	15.2 ± 9.7 (3.5–36.4)
	110–119	2	111.5 ± 0.7	61 ± 4.2 (58–64)	126.4 ± 21.1 (111.4–141.3)	34.5 ± 3.5 (32–37)	119.5 ± 44.5 (88–151)	13.7± 11.5 (5.621.8)
Pearson correlation One-way ANOVA				r = 0.0789 *p* < 0.001	r = 0.068 *p* < 0.001	r = 0.857 *p* < 0.001	r = 0.331 *p* < 0.001	r = 0.535 *p* < 0.001
CC	<7	9.0	5.9 ± 2.3	67.6 ± 17.0 (51–88)	75.0 ± 40.2 (0–131.6)	23.4 ± 0.7 (22–24)	42.9 ± 26.1 (15–98)	1.0 ± 0.6 (0.3–2.2)
	10–19	104.0	16.1 ± 2.4	57.9 ± 14.6 (31–91)	94.0 ± 42.5 (11.9–184.6)	25.8 ± 0.7 (24–28)	44.5 ± 23.1 (10–109)	1.4 ± 0.7 (0.3–2.8)
	20–29	585.0	25.7 ± 2.7	55.7 ± 12.9 (19–91)	101.1 ± 38.4 (0–222.7)	26.2 ± 0.5 (24–28)	60.5 ± 26.6 (12–184)	2.0 ± 0.8 (0.3–4.7)
	30–39	1949.0	35.4 ± 2.8	55.2 ± 12.5 (19–88)	104.0 ± 39.6 (0–234.2)	27.5 ± 0.7 (25–30)	72.0 ± 38.1 (10–327)	2.9 ± 1.2 (0.4–8.6)
	40–49	4143.0	44.9 ± 2.8	54.3 ± 11.9 (19–87)	99.0 ± 37.3 (0–228.6)	28.1 ± 0.7 (25–33)	78.9 ± 49.0 (8–442)	3.4 ± 1.7 (0.3–18.3)
	50–59	4882.0	54.4 ± 2.8	53.8 ± 10.1 (19–90)	99.0 ± 34.9 (0–229.8)	29.4 ± 0.7 (26–34)	93.0 ± 63.6 (8–470)	4.5 ± 2.4 (0.4–22.5)
	60–69	2837.0	63.8 ± 2.8	52.5 ± 9.0 (19–86)	97.1 ± 35.4 (0–231.2)	30.3 ± 1.0 (26–38)	124.8 ± 97.6 (8–574)	6.4 ± 4.2 (0.3–30.4)
	70–79	865.0	73.3 ± 2.6	51.3 ± 8.4 (20–83)	90.4 ± 36.2 (0–217.0)	30.8 ± 1.3 (26–34)	146.9 ± 99.1 (8–574)	8.9 ± 5.8 (0.3–35.5)
	80–89	178.0	83.2 ± 2.6	51.0 ± 7.2 (35–78)	82.4 ± 36.7 (0–177.5)	32.2 ± 0.9 (27–34)	159.6 ± 88.9 (45–441)	11.1 ± 6.2 (1.9–31.6)
	90–99	25.0	92.0 ± 2.1	42.9 ± 11.3 (19–58)	59.2 ± 46.2 (0–139.0)	32.0 ± 2.4 (27–34)	144.7 ± 106.5 (45–509)	10.6 ± 8.1 (2.0–34.4)
	100–109	8.0	102.6 ± 2.6	51.1 ± 10.1 (37–64)	57.1 ± 47.9 (0–123.9)	31.4 ± 3.1 (26–34)	84.5 ± 26.7 (50–127)	6.1 ± 3.0 (2.0–9.9)
	110–119	4.0	112.0 ± 2.0	42.5 ± 26.0 (20–66)	44.1 ± 52.7 (0–104.9)	31.8 ± 6.7 (26–38)	149.8 ± 156.4 (50–379)	14.8± 16.4 (2.1–36.6)
Pearson correlation One-way ANOVA				r = 0.116 *p* < 0.001	r = 0.0801 *p* < 0.001	r = 0.847 *p* < 0.001	r = 0.329 *p* < 0.001	r = 0.515 *p* < 0.001

CC = craniocaudal; MLO = mediolateral oblique; ANOVA, statistically significant differences at *p* < 0.05.

**Table 4:** The present study’s MGD DRLs in line with local/regional and national reported DRLs.

CBT (mm)	Present Work		References		
	MGD DRL (mGy)		MGD DRL (mGy)/View		
	CC	MLO	CC	MLO	All View
<7	0.94	0.44			
10–19	1.08	1.17			
20–29	1.11	1.17			0.97 (CBT:20–29 mm) [9]
30–39	1.3	1.32	1.6 (CBT:36 mm) [21]; 3.48 (CBT:36 mm) [23];		1.12 (CBT:30–39 mm) [9]
40–49	1.31	1.38		2.4 (CBT: 45 mm) [21] 2.03 (CBT: 44 mm) [23]	1.31 (CBT:40–49 mm) [9]
50–59	1.61	1.75	1.68 (CBT: 50.9 mm) [22] 2.5 (CBT: 50–60 mm) [24] 1.6 (CBT: 50 mm) [25]	2.25 (CBT: 58.9 mm) [22] 1.8 (CBT: 50 mm) [25]	1.65 (CBT:50–59 mm) [9] 1.7 (CBT:50 mm) [25]
60–69	2.11	2.25	1.23 (CBT: 60–69 mm) [26]	1.32 (CBT: 60–69 mm) [26]	2.35 (CBT: 60–69 mm) [9]
70–79	2.81	2.80			2.08 (CBT: 70–79 mm) [9]
80–89	3.2	3.38			2.34 (CBT: 80–89 mm) [9]
90–99	2.54	4.02			2.63 (CBT: 90–99 mm) [9]
100–109	1.63	4.40			3.31 (CBT:100–110 mm) [9]

**Table 5:** MGD values for various CBT ranges, comparison with European DRLs.

CBT Range (mm)	MGD Typical Value (mGy)		MGD European DLR (mGy)	
	CC	MLO	Acceptable	Achievable
20–29	0.92	0.94	<1.0	<0.6
30–39	1.07	1.09	<1.5	<1.0
40–49	1.09	1.15	<2.0	<1.6
50–59	1.30	1.40	<2.5	<2.0
60–69	1.63	1.74	<3.0	<2.4
70–79	2.05	2.04	<4.5	<3.6
80–89	2.34	2.40	/	/
90–99	2.15	2.87	<6.5	<5.1

## Data Availability

The data presented in this study are available upon request from the corresponding author.

## Abbreviations

The following abbreviations are used in this manuscript:

MGD	Mean glandular dose
DRL	Diagnostic reference level
CC	Craniocaudal
MLO	Mediolateral oblique
CBT	Compressed breast thickness
kVp	Peak kilovoltage
mAs	Tube current
IQR	Interquartile range
kVp	Peak kilovoltage
mAs	Current
